# Analysis of the Morpho-Geometrical Changes of the Root Canal System Produced by TF Adaptive vs. BioRace: A Micro-Computed Tomography Study

**DOI:** 10.3390/ma14030531

**Published:** 2021-01-22

**Authors:** Loai Alsofi, Muhannad Al Harbi, Martin Stauber, Khaled Balto

**Affiliations:** 1 Department of Endodontics, Faculty of Dentistry, King Abdulaziz University, Jeddah 21589, Saudi Arabia; Mohannada@moh.gov.sa (M.H.); kbalto@kau.edu.sa (K.B.); 2 Ministry of Health, Al Thaghr Hospital, Al Thaghr, Jeddah 22361, Saudi Arabia; 3 SCANCO Medical AG, 8306 Brüttisellen, Switzerland; martin.stauber@gratxray.com

**Keywords:** micro-computed tomography, nickel-titanium instruments, root canal preparation, endodontic drills, TF Adaptive, iRace

## Abstract

We aimed to analyze the morpho-geometric changes of the root canal system created by two rotary systems (TF Adaptive and BioRace) using micro-CT technology. Two concepts of rotary file system kinematics, continuous rotation and adaptive kinematics, were used in root canal preparation. Twenty mandibular molars (n = 20) were selected with the following criteria: the teeth have mesial roots with a single and continuous isthmus connecting the mesiobuccal and mesiolingual canals (Vertucci’s Type I configuration) and distal roots with independent canals. Teeth were scanned at a resolution of 14 μm. Canals were divided equally into two groups and then enlarged sequentially using the BioRace system and TF Adaptive system according to manufacturer protocol. Co-registered images, before and after preparation, were evaluated for morphometric measurements of canal surface area, volume, structure model index, thickness, straightening, and un-instrumented surface area. Before and after preparation, data were statistically analyzed using a paired sample *t*-test. After preparation, data were analyzed using an unpaired sample test. The preparation by both systems significantly changed canal surface area, volume, structure model index, and thickness in both systems. There were no significant differences between instrument types with respect to these parameters (*p* > 0.05). TF Adaptive was associated with less straightening (8% compared with 17% for BioRace in the mesial canal, *p* > 0.05). Both instrumentation systems produced canal preparations with adequate geometrical changes. BioRace straightened the mesial canals more than TF Adaptive.

## 1. Introduction

Three-dimensional cleaning and shaping of the root canal system of the teeth is the key for three-dimensional obturation [1,2]. Several nickel–titanium (NiTi) instrument systems have been introduced on the market. These instruments along with the different irrigation solutions facilitate the biomechanical cleaning and shaping of the root canal system. NiTi rotary files may undergo fatigue without showing signs of deterioration on the flutes [3,4,5]. Most companies are trying to develop novel manufacturing technologies to overcome the inherent deficiencies. Such new technologies include M-wire, the newly introduced controlled memory, and thermal technology [6,7,8,9,10]. Alteration to the root canal anatomy, particularly in the apical third of the root canal space, is another key shortcoming of the current instrumentation systems [7,8]. This may create space inside the root canal, which may harbor bacteria and other microbes.

All NiTi rotary file systems available on the market are manufactured using the machine grinding technique, except two, which are twisted files (TF) and TF Adaptive systems (Kerr, Brea, CA, USA). The Twisted File Adaptive system (TF Adaptive) is used in combination with continuous rotation and reciprocation (Kerr, Brea, CA, USA). Reports indicate that reciprocating files result in a marked improvement in cyclic fatigue resistance [11]. The file operates in continuous rotation when minimal pressure is applied, and in reciprocal mode when it engages dentin and the load is increased. Manufacturers argue that this adaptive technology and twisted file design enhances flexibility and allows files to adjust to intracanal torsional stress.

The BioRace system (FKG Dentsaire SA, La Chaux-de-Fonds, Switzerland) is a simplified version of the original Race system (FKG Dentsaire SA). It has active cutting regions, which are electrochemically polished, and twisted areas with alternating cutting edges [12]. BioRace files are another promising option to improve clinical performance [13]. We aimed to evaluate and compare, in an ex vivo model, the shaping ability of adaptive reciprocation kinematics and continuous rotation instrumentation movement using TF Adaptive files and BioRace files, respectively, using micro-computed tomography (micro-CT).

The null hypothesis of the study was that there is no statistically significant difference in the morpho-geometric changes produced in root canals by BioRace and TF Adaptive system.

## 2. Materials and Methods

### 2.1. Experimental Teeth Selection

After local research ethics committee approval from King Abdulaziz University, Jeddah, Saudi Arabia (protocol no. 2016/145), one hundred extracted human mandibular first molars were obtained from a pool of teeth. Preapical radiographs were taken from buccolingual and mesiodistal views to ensure they had noncalcified canals. Teeth were stored in 0.1% thymol solution at 4 °C [14]. Inclusion criteria were: teeth with two mesial canals and one distal canal, teeth that had completely formed roots, had both mesial canals connected by a single and continuous isthmus (Vertucci type II configuration), and had a root curvature range of 15°–20° in both the mesiodistal and buccolingual directions. Exclusion criteria were carious teeth and teeth with root resorption or visible cracks. With these criteria, twenty human mandibular first molars were included in this study. Teeth were cleaned using Kavo ultrasonic peizo scaler (Kavo, Biberach an der Riss, Germany) and inspected under magnification (20×) using a dental operating microscope (Zeiss, Oberkochen, Germany)

### 2.2. Teeth Preparation

The twenty teeth were randomly divided into two groups (10 teeth in each group): group A (TF, n = 20 canals) and group B (BioRace, n = 20 canals). The teeth were mounted to a special-purpose sample holder. The tips of the roots were covered with utility wax to create a closed-end system and to prevent the intrusion of the rubber base material into the apical part of the canal. Standard access cavity preparation was performed using a diamond-coated bur [15]. Working length was determined using a size 15 K-file with the aid of periapical radiographs [16]. In group A, ten first mandibular molars were prepared using the TF Adaptive rotary system (Kerr, Brea, CA, USA) according to the manufacturer’s instructions after establishing the glide path to full working length using a size 15 K-file. Teeth were prepared with TF Adaptive small canal system SM1 20/0.4, SM2 25/0.6, and SM3 35/0.4 to full working length using an elements motor (Kerr, Brea, CA, USA) at the installed recommended setting for the TF Adaptive in adaptive motion. Standard irrigation, as described above, was performed between each file. The rotary system files were used once per tooth. Each canal was dried with absorbent paper (35/4%; Dentsply Maillefer). Each file was carefully cleaned of debris after the preparation of each root canal using Korsolex Endo-Cleaner [17]. In group B, ten first mandibular molars were prepared using the BioRace rotary system (FKG, La Chaux-de-Fonds, Switzerland) according to the manufacturer’s instructions after establishing the glide path to full working length using a size 15 K-file (Dentsply Maillefer, Ballaigues, Switzerland). Teeth were prepared with R1 15/0.6, R2 25/0.4, R3 30/0.4, and BioRace 35/0.4 to full working length using an elements motor (Kerr, Brea, CA, USA) with 600 rpm and 1.5 N/cm torque in continuous rotation [18].

Irrigation was performed using a 30 gauge side-vented needle (Ultradent, South Jordan, UT, USA) with a 5 mL syringe. The needle was inserted up to 1 mm shorter than the working length. The total amount of fluid for each canal was 5 mL of 5.25% NaOCl and 2 mL of 17% Ethylenediaminetetraacetic acid (EDTA) as a final flush after canal preparation. Teeth were irrigated with 1 mL of 5.25% NaOCl for each step of canal preparation as follows: irrigation with 1 mL of 5.25% NaOCl before instrumentation, between each instrument, and after instrumentation. A final flush was conducted with 2 mL of 17% EDTA [19].

Standard irrigation, as described above, was performed between each file. The rotary system files were used once per tooth. Each canal was dried with absorbent paper (35/4%; Dentsply Maillefer). Each file was carefully cleaned of debris after the preparation of each root canal using Korsolex Endo-Cleaner.

### 2.3. Micro-CT Analysis

The teeth were embedded in a special sample holder to ensure reproducible positioning for the repetitive measurements. The specimens were scanned with a μCT 100 (Scanco Medical AG, Brüttisellen, Switzerland) at an energy of 90 kVp, an intensity of 88 μA, and an integration time of 500 ms per projection. The data were reconstructed to an isotropic voxel size of 14 μm using a filtered back-projection algorithm. These settings were used for all base and follow-up measurements.

The outer contour of each tooth was generated automatically using a special-purpose algorithm. This outer contour was limited to a region that started at 50 slices above the slice where the root canals merged, and ended at the tip of the root. This outer contour was used for separating the background from the root canal, which was important for teeth where the root was cracked. Within this outer contour, the root canals could be extracted using global segmentation procedures.

Although the teeth were embedded, corresponding follow-up measurements did not fit perfectly. For this reason, a rigid registration algorithm was used to register the gray-level images. The main challenge with this procedure is that there are not many internal structures or features that allow for accurate registration. Therefore, the outer shape and gray-level intensities were the most significant features that could be used for the registration. With this registration, an accurate result could be achieved. Qualitative assessment was accomplished by the superimposition of constructed three-dimensional images showing the un-instrumented canal in green and the instrumented canal in red. Cross-section images perpendicular to the root canal were extracted and compared for each phase of the experiment. Volume and surface area of root canals were evaluated before and after instrumentation, and the changes were calculated as the difference between the pre- and post-instrumentation scores. The thickness was calculated along the canal using distance transformation techniques [20]. The structure model index (SMI) was calculated to determine the flatness of the root canal [21]. The centers of gravity of the canal were calculated slice-wise and connected by fitting a line, which was further used to calculate the curvature of the root canal [20]. Straightening is expressed as the difference between the post-instrumentation canal curvature (fitted line) and the initial curvature (in %). The un-instrumented surface area was calculated by evaluating the superimposed images through matching images of the surface area of the canal before and after preparation. A key assumption, in this case, was that surface voxels remained in the same places before and after preparation.

### 2.4. Statistical Analysis

The Shapiro–Wilk normality test was used to test all baseline measurements from mesial and distal roots. After instrumentation, we compared data from the baseline and data from after instrumentation measurements of the two file systems. Statistical analysis was performed using a paired sample *t*-test for normally distributed data (before and after instrumentation). An unpaired sample *t*-test was used for normally distributed data between nonparametric Mann–Whitney test for non-normally distributed data at a *p*-value of 0.05. Prism 8 software (Version 8, GraphPad Software, La Jolla, CA, USA) was used for analysis.

## 3. Results

All baseline parameters of mesial and distal roots showed normal distribution except for canal volume of both mesial and distal roots. Normally distributed data included structure model index (SMI), surface area, and the thickness of the canal. Table 1 shows μCT data before and after the preparation of the mesial canal for both TF Adaptive and BioRace systems. Table 2 shows μCT data before and after the preparation of the distal canal for both TF Adaptive and BioRace systems. The indices shown are as follows: volume, surface area, structural model index, average root canal thickness, and unprepared surface area. Both systems resulted in a significant change in root canal parameters when comparing before and after data in both mesial and distal canals.

In mesial canals, 36–42% of the root canal surface was unprepared. The BioRace group showed slightly higher untreated voxels than the TF group. This indicated that the TF group touched more surface area in the mesial canals (Table 1). In the distal canal, the after preparation un-instrumented canal surface area ranged from 46–52%. The TF group showed slightly more untreated voxels in the distal canal, indicating that BioRace group touched more surface area in the distal canal. However, differences were not statistically significant between the groups, nor in the mesial or the distal canals (Table 2).

**Figure 1:**
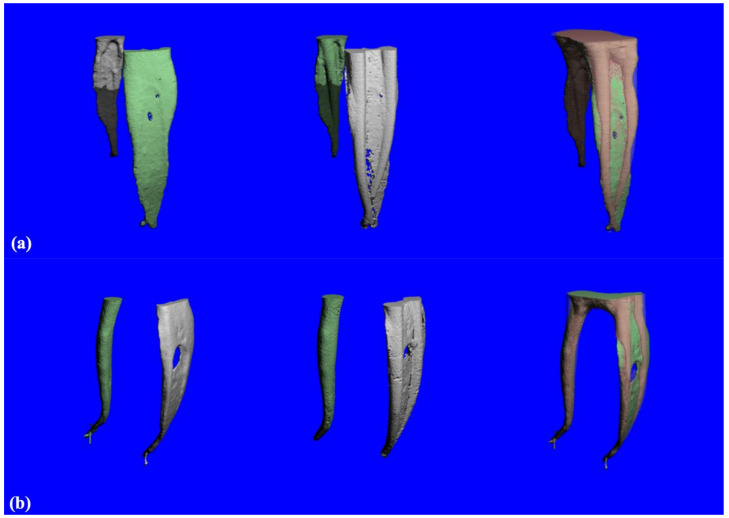
(**a**) 3D-constructed images from the TF Adaptive group of root canal system before (left) and after (middle) instrumentation and superimposed image (right) from the mesial. (**b**) 3D constructed images from BioRace group of root canal system before (left) and after (middle) instrumentation and superimposed image (right) from the mesial. Green indicates un-instrumented areas while red indicates instrumented areas.

Figure 1a shows 3D-constructed images of the root canal system prepared using TF files before (left) and after (middle) instrumentation, as well as a superimposed image (right) from the mesial view. Figure 1b shows the 3D-constructed images of the root canal system prepared using BioRace system before (left) and after (middle) instrumentation, as well as a superimposed image (right) from the mesial view.

**Figure 2:**
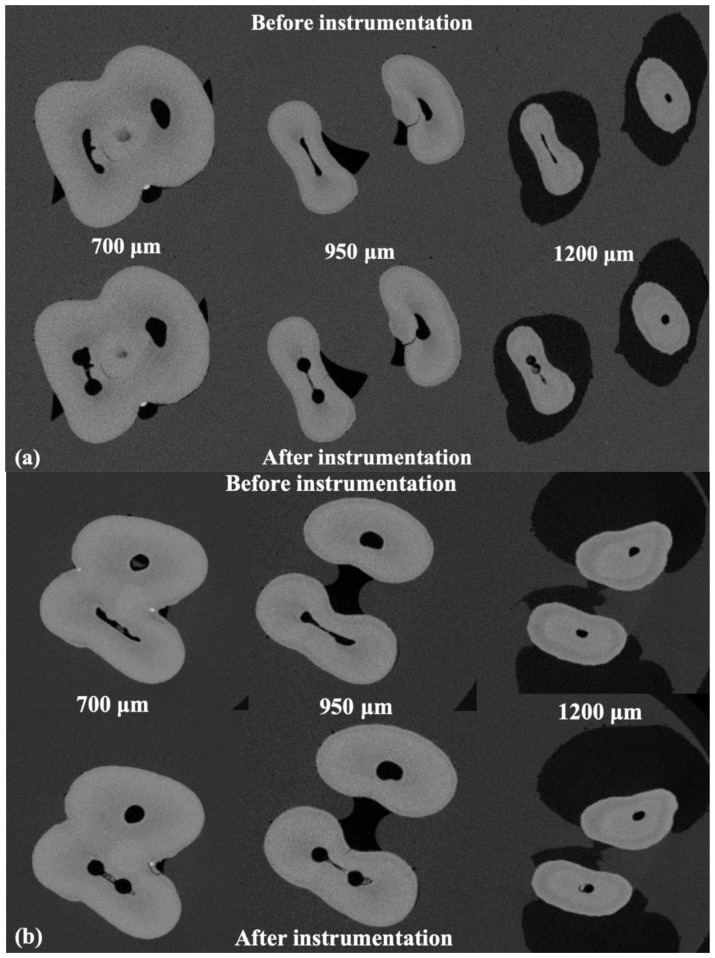
(**a**) Cross-section images from different level slices 700 μm (top), 950 μm (middle), and 1200 μm (bottom) obtained from micro-CT image before (images on the left) and after (images on the right) root canal preparation using TF system. (**b**) Cross-section images from deferent level slices 700 μm (top), 950 μm (middle), and 1200 μm (bottom) obtained from micro-CT image before (images on the left) and after (images on the right) root canal preparation using BioRace system.

Figure 2a shows cross-section images from different levels: 700 μm (top), 950 μm (middle), and 1200 μm (bottom) obtained from micro-CT image before (images on the left) and after (images on the right) root canal preparation using TF system. Figure 2b shows cross-section images from different level slices 700 μm (top), 950 μm (middle), and 1200 μm (bottom) obtained from micro-CT image before (images on the left) and after (images on the right) root canal preparation using BioRace system preparation. The relative degrees of canal straightening in BioRace and TF Adaptive groups were 17.56% ± 10.7% and 8.87% ± 6.84% in mesial canals, respectively, with no significant differences between instrument type (*p* > 0.5). In the distal canal, there was no significant difference in canal straightening for BioRace and TF Adaptive groups, 12.1% ± 12.9%, and 9.6% ± 5.6% respectively.

## 4. Discussion

The main objective of root canal preparation is to create a tapered shape from apical to coronal areas while maintaining the original shape and keeping the apical diameter as small as possible [22]. This procedure may result in several preparation errors, such as ledge formation, perforation, canal transportation, file separation, elbow, apical zip, and canal blockage [23]. BioRace and TF Adaptive systems were designed to improve the canal shape while reducing unwanted procedural side effects.

When comparing the morpho-geometric changes after root canal preparation, it is important to have apical preparation diameters and similar tapers [24]. In this study, we compared the effects of two root canal instrumentation systems on the morpho-geometric changes. We chose BioRace and TF Adaptive systems because of their similarity in cross-section and instrument design. The only differences between these two devices are the kinematics and manufacturing process, which could be one of the limitations of this study. A recent study by Alghamdi et al. compared the effect of thermomechanical treatment of two rotary systems with similar design on the morpho-geometric morphology of prepared root canals [25].

In this study, the morpho-geometric changes were quantitatively analyzed using a set of measures such as the surface area, volume, thickness, and SMI. Furthermore, the mean values of the entire length of the canal’s three-dimensional geometry were calculated [20]. We found that changes in canal geometry after instrumentation depend more on the canal type rather than the technique. This adds another limitation to the study, which is the variability in teeth anatomy between experimental groups.

In the mesial canals, instrumentation changed the geometry of the root canals. With the BioRace system, significant changes in volume and SMI were found. The significant change in volume could be explained by BioRace files working in continuous rotation only and thus lack the adaptive counterclockwise motion. This is especially pronounced in narrow canals such as the mesial canals in lower molars. The changes in SMI with BioRace indicated that it tends to change the general geometry of the root canal by transforming the original flat canals to conical ones. This indicated that the BioRace system left larger and more rounded canals after preparation than TF Adaptive in the mesial canal.

In the distal canals, the surface area and SMI were slightly increased, without being statistically significant, with both systems. This indicated that surface area and canal roundness in the distal canals were less affected than in mesial canals. This may be explained by anatomical variations and the size of the canal, which is much larger compared to mesial canals. Therefore, the instrumentation method has less influence on the resulting canal shape.

The mean untouched canal walls ranged from 36–42%. Both systems were unable to clean the root canal completely, which agrees with previous studies [8,21,26,27]. A comparable study by Velozo et al. showed that XP-Endo Shaper and ProTaper Next have similar canal-shaping ability when used in oval canals in mandibular incisors. All preparation parameters (volume, surface area, structure model index, and untouched walls) were significantly increased with no statistically significant difference between the two systems [28]. However, in our study, the tissue volume removed from the canals was higher than in other reports with similar methodology (5–30%) [29,30,31]. This may be due to the complex anatomy of the selected teeth, which may have a greater effect than the instrumentation techniques [20]. However, no significant difference was found between the two systems. If the amount of dentin removed was <34 µm, it would not have been registered [20] in this study; however, removing <34 µm of dentin was not sufficient because microorganisms can penetrate up to 150 µm inside the dentinal tubules [32].

NiTi rotary instruments tend to maintain the original canal curvature, even in extremely curved canals [33,34]. In this study, canal curvature was evaluated by fitting a line through the centers of gravity of each slice along the *z*-axis. This line was calculated for the un-instrumented and instrumented canals to calculate the straightening. In agreement with previous studies [35,36,37], we found that TF Adaptive maintained the original canal curvature better than BioRace in the mesial canal. This may be due to its alloy martensitic nature and unique adaptive motion. As the distal canals are much wider, they have less resistance; thus, the adaptive motion has little effect. Therefore, we found no difference between the two techniques in distal canals. However, further studies are needed to better evaluate the effect of adaptive motion on the straightening of the canal.

Excessive removal of dentin may lead to root fracture [37,38,39]. However, if the instrument is well-centered, more dentin can be preserved and the stability of the roots can be maintained [1]. In our study, the dentin was much better preserved than in studies performed with conventional instruments. This may be explained by the similar design of the two systems. The effect of the heat treatment of the NiTi in the TF group did not produce a superior result compared to BioRace in terms of remaining thickness, which is a conventional NiTi. It is expected that heat-treated NiTi systems would produce superior results to conventional NiTi systems with a similar design. This can be attributed to their plastic deformation, which can improve the cutting efficiency of the cutting edges during instrumentation, as mentioned in previous studies [31,40]. The limitations of our study include the different design features between both systems and the selection of the teeth based on clinical criteria and conventional radiographs rather than micro-CT evaluation.

## 5. Conclusions

Both rotary systems produced canal preparations with adequate geometrical changes. The BioRace system tended to produce more changes in canal geometry, volume, and more straightening, whereas the TF Adaptive system did not induce significant changes to the original canal curvature and geometry as much as BioRace. Neither of the two systems could touch all the canal walls.

## Figures and Tables

**Table 1:** Morphometric indices before and after instrumentation of mesial canals.

Parameters		BioRace n = 20 Mean ± SD	*p* **	TF Adaptive n = 20 Mean ± SD	*p* **	*p* *
Volume	Before (mm^3^)	4.18 ± 1.48		5.12 ± 2.62		0.338
	After (mm^3^)	5.84 ± 1.13		6.67 ± 2.57		0.365
	Increase (Δ%)	1.67 ± 0.74	<0.001 **	1.56 ± 1.07	0.001 **	0.969
Surface Area	Before (mm^2^)	42.37 ± 12.56		44.72 ± 17.26		0.732
	After (mm^2^)	46.99 ± 10.69		49.43 ± 17.50		0.711
	Increase (Δ%)	4.62 ± 6.07	0.039 **	4.71 ± 3.97	0.005 **	0.789
Structural Model Index (SMI)	Before	1.80 ± 1.07		1.95 ± 0.91		0.739
	After	2.51 ± 1.24		2.10 ± 0.78		0.385
	Increase (Δ%)	0.71 ± 0.88	0.030 **	0.15 ± 0.52	0.384	0.195
Thickness	Before (mm)	0.321 ± 0.14		0.375 ± 0.14		0.394
	After (mm)	0.53 ± 0.09		0.53 ± 0.07		0.915
	Increase (Δ%)	0.21 ± 0.099	<0.001 **	0.15 ± 0.09	0.001 **	0.195
Unprepared Area	Static Voxels	80,468.20 ± 35		67,006.70 ± 22		0.323
	After (%)	42 ± 15%		36 ± 14%		0.405

* *p*-value for significance between TF Adaptive and BioRace. ** *p*-value for significance between before and after instrumentation data for the same instrument.

**Table 2:** Morphometric indices before and after instrumentation of distal canals.

Parameters		BioRace n = 20 Mean ± SD	*p* **	TF Adaptive n = 20 Mean ± SD	*p* **	*p* *
Volume	Before (mm^3^)	5.85 ± 1.86		7.58 ± 4.59		0.283
	After (mm^3^)	7.25 ± 1.97		8.22 ± 4.46		0.534
	Increase (Δ%)	1.40 ± 0.88	0.001 **	0.64 ± 0.66	0.014 **	0.043
Surface Area	Before (mm^2^)	48.71 ± 14.99		47.55 ± 25.22		0.902
	After (mm^2^)	51.86 ± 14.78		49.93 ± 29.59		0.856
	Increase (Δ%)	3.14 ± 4.67	0.062	2.38 ± 5.92	0.236	0.751
SMI	Before	1.04 ± 1.32		1.28 ± 0.87		0.638
	After	1.50 ± 1.21		1.46 ± 1.14		0.942
	Increase (Δ%)	0.46 ± 0.93	0.154	0.18 ± 1.04	0.595	0.536
Thickness	Before (mm)	0.38 ± 0.16		0.47 ± 0.12		0.193
	After (mm)	0.52 ± 0.10		0.57 ± 0.11		0.288
	Increase (Δ%)	0.14 ± 0.09	0.001 **	0.10 ± 0.07	0.001 **	0.358
Unprepared Area	Static Voxels	86,191.50 ± 42,415.72		100,673.80 ± 40,002.76		0.442
	After (%)	46 ± 22		52 ± 17		0.551

* *p*-value for significance between TF Adaptive and BioRace. ** *p*-value for significance between before and after instrumentation data for the same.

## Data Availability

The data presented in this study are available on request from the corresponding author. The data are not publicly available due to intellectual reasons.

## References

[1] Hulsmann M., Peters O.A., Dummer P.M. (2005). Mechanical preparation of root canals: Shaping goals, techniques and means. Endod. Top..

[2] Schilder H. (1974). Cleaning and shaping the root canal. Dent. Clin. N. Am..

[3] Aydin C., Inan U., Tunca Y.M. (2010). Comparison of cyclic fatigue resistance of used and new RaCe instruments. Oral Surg. Oral Med. Oral Pathol. Oral Radiol. Endod..

[4] Aydin C., Inan U., Yasar S., Bulucu B., Tunca Y.M. (2008). Comparison of shaping ability of RaCe and Hero Shaper instruments in simulated curved canals. Oral Surg. Oral Med. Oral Pathol. Oral Radiol. Endod..

[5] Sattapan B., Nervo G.J., Palamara J.E., Messer H.H. (2000). Defects in rotary nickel-titanium files after clinical use. J. Endod..

[6] Bidar M., Moradi S., Forghani M., Bidad S., Azghadi M., Rezvani S., Khoynezhad S. (2010). Microscopic evaluation of cleaning efficiency of three different nickel-titanium rotary instruments. Iran. Endod. J..

[7] Capar I.D., Arslan H., Akcay M., Ertas H. (2014). An in vitro comparison of apically extruded debris and instrumentation times with ProTaper Universal, ProTaper Next, Twisted File Adaptive, and HyFlex instruments. J. Endod..

[8] Capar I.D., Ertas H., Ok E., Arslan H., Ertas E.T. (2014). Comparative study of different novel nickel-titanium rotary systems for root canal preparation in severely curved root canals. J. Endod..

[9] Shen Y., Zhou H.M., Zheng Y.F., Campbell L., Peng B., Haapasalo M. (2011). Metallurgical characterization of controlled memory wire nickel-titanium rotary instruments. J. Endod..

[10] Alapati S.B., Brantley W.A., Iijima M., Clark W.A., Kovarik L., Buie C., Liu J., Ben Johnson W. (2009). Metallurgical characterization of a new nickel-titanium wire for rotary endodontic instruments. J. Endod..

[11] Jin S.Y., Lee W., Kang M.K., Hur B., Kim H.C. (2013). Single file reciprocating technique using conventional nickel-titanium rotary endodontic files. Scanning.

[12] Saber S.E., Nagy M.M., Schäfer E. (2015). Comparative evaluation of the shaping ability of ProTaper Next, iRaCe and Hyflex CM rotary NiTi files in severely curved root canals. Int. Endod. J..

[13] Vadhana S., SaravanaKarthikeyan B., Nandini S., Velmurugan N. (2014). Cyclic fatigue resistance of RaCe and Mtwo rotary files in continuous rotation and reciprocating motion. J. Endod..

[14] Strawn S.E., White J.M., Marshall G.W., Gee L., Goodis H.E., Marshall S.J. (1996). Spectroscopic changes in human dentine exposed to various storage solutions--short term. J. Dent..

[15] Yamamura B., Cox T.C., Heddaya B., Flake N.M., Johnson J.D., Paranjpe A. (2012). Comparing canal transportation and centering ability of endosequence and vortex rotary files by using micro-computed tomography. J. Endod..

[16] Turkistani A.K., Gomaa M.M., Shafei L.A., Alsofi L., Majeed A., AlShwaimi E. (2019). Shaping Ability of HyFlex EDM and ProTaper Next Rotary Instruments in Curved Root Canals: A Micro-CT Study. J. Contemp. Dent. Pract..

[17] Gambarini G., Testarelli L., De Luca M., Milana V., Plotino G., Grande N.M., Rubini A.G., Al Sudani D., Sannino G. (2013). The influence of three different instrumentation techniques on the incidence of postoperative pain after endodontic treatment. Ann. Stomatol. (Roma).

[18] Bonaccorso A., Cantatore G., Condorelli G.G., Schafer E., Tripi T.R. (2009). Shaping ability of four nickel-titanium rotary instruments in simulated S-shaped canals. J. Endod..

[19] Paque F., Zehnder M., De-Deus G. (2011). Microtomography-based comparison of reciprocating single-file F2 ProTaper technique versus rotary full sequence. J. Endod..

[20] Peters O.A., Laib A., Göhring T.N., Barbakow F. (2001). Changes in root canal geometry after preparation assessed by high-resolution computed tomography. J. Endod..

[21] Peters O.A., Schonenberger K., Laib A. (2001). Effects of four Ni-Ti preparation techniques on root canal geometry assessed by micro computed tomography. Int. Endod. J..

[22] Thompson S.A., Dummer P.M. (2000). Shaping ability of Hero 642 rotary nickel-titanium instruments in simulated root canals: Part 2. Int. Endod. J..

[23] Al-Omari M.A., Dummer P.M. (1995). Canal blockage and debris extrusion with eight preparation techniques. J. Endod..

[24] Bergmans L., Van Cleynenbreugel J., Beullens M., Wevers M., Van Meerbeek B., Lambrechts P. (2003). Progressive versus constant tapered shaft design using NiTi rotary instruments. Int. Endod. J..

[25] Alghamdi A., Alsofi L., Balto K. (2020). Effects of a Novel NiTi Thermomechanical Treatment on the Geometric Features of the Prepared Root Canal System. Materials.

[26] Gergi R., Osta N., Bourbouze G., Zgheib C., Arbab-Chirani R., Naaman A. (2015). Effects of three nickel titanium instrument systems on root canal geometry assessed by micro-computed tomography. Int. Endod. J..

[27] Zhao D., Shen Y., Peng B., Haapasalo M. (2014). Root canal preparation of mandibular molars with 3 nickel-titanium rotary instruments: A micro-computed tomographic study. J. Endod..

[28] Velozo C., Silva S., Almeida A., Romeiro K., Vieira B., Dantas H., Sousa F., De Albuquerque D.S. (2020). Shaping ability of XP-endo Shaper and ProTaper Next in long oval-shaped canals: A micro-computed tomography study. Int. Endod. J..

[29] Peters O.A., Boessler C., Paqué F. (2010). Root canal preparation with a novel nickel-titanium instrument evaluated with micro-computed tomography: Canal surface preparation over time. J. Endod..

[30] Paque F., Balmer M., Attin T., Peters O.A. (2010). Preparation of oval-shaped root canals in mandibular molars using nickel-titanium rotary instruments: A micro-computed tomography study. J. Endod..

[31] Gagliardi J., Versiani M.A., de Sousa-Neto M.D., Plazas-Garzon A., Basrani B. (2015). Evaluation of the Shaping Characteristics of ProTaper Gold, ProTaper NEXT, and ProTaper Universal in Curved Canals. J. Endod..

[32] Sen B.H., Piskin B., Demirci T. (1995). Observation of bacteria and fungi in infected root canals and dentinal tubules by SEM. Endod. Dent. Traumatol..

[33] Hülsmann M., Gressmann G., Schäfers F. (2003). A comparative study of root canal preparation using FlexMaster and HERO 642 rotary Ni-Ti instruments. Int. Endod. J..

[34] Versümer J., Hülsmann M., Schäfers F. (2002). A comparative study of root canal preparation using Profile .04 and Lightspeed rotary Ni-Ti instruments. Int. Endod. J..

[35] Aminsobhani M., Razmi H., Nozari S. (2015). Ex Vivo Comparison of Mtwo and RaCe Rotary File Systems in Root Canal Deviation: One File Only versus the Conventional Method. J. Dent. (Tehran).

[36] Qiu N., Wang C.Y., Liu Y.F., Yu X.Q., Xue M. (2016). Comparison of the shaping ability of three Ni-Ti rotary instruments in the preparation of simulated curved root canals. Shanghai Kou Qiang Yi Xue.

[37] Ordinola-Zapata R., Bramante C.M., Duarte M.A., Cavenago B.C., Jaramillo D., Versiani M.A. (2014). Shaping ability of reciproc and TF adaptive systems in severely curved canals of rapid microCT-based prototyping molar replicas. J. Appl. Oral Sci..

[38] Kishen A. (2006). Mechanisms and risk factors for fracture predilection in endodontically treated teeth. Endod. Top..

[39] Tang W., Wu Y., Smales R.J. (2010). Identifying and reducing risks for potential fractures in endodontically treated teeth. J. Endod..

[40] Shen Y., Coil J.M., Zhou H., Zheng Y., Haapasalo M. (2013). HyFlex nickel-titanium rotary instruments after clinical use: Metallurgical properties. Int. Endod. J..

